# Bilateral atrophic squirrhus of breast in neglected breast cancer: case report

**DOI:** 10.11604/pamj.2014.19.316.2821

**Published:** 2014-11-26

**Authors:** Doulira Louati, Khaled Trigui, Donia Abid, Salma Kammoun, Manel Dermech, Kais Chaabane, Habib Amouri

**Affiliations:** 1 Obstetrics and Gynecology Department, Hédi Chaker Academic Hospital, Sfax, Tunisia

**Keywords:** atrophic squirrhus carcinoma, bilateral breast cancer, lymphoedema

## Abstract

The atrophic squirrhus carcinoma is an advanced form of breast cancer, which is most often neglected by patients. These days it has become very rare. The bilaterality of this form is even more exceptional. We present a case of atrophic squirrhus breast cancer of a 58 years old woman, rural origin, which is particular for its bilaterality and rapid evolution causing the death after 22 months from the first abnormal functional sign.

## Introduction

The advanced malignant tumours of the breast are frequent in our country. Indeed, they represent 15 to 20% of the mammary carcinomas. The atrophic squirrhe of the breast is an entity which becomes currently exceptional, in that, its history is characterised by the disappearance of the glandular areas, which is responsible for an atrophy of the mammary gland with retraction of the tumour. It especially touches aged women. We study a case of atrophic squirrhe of the breast treated in the Obstetrics and Gynecology Department of university hospital Hèdi Chaker Sfax /Tunisia. The cancer is particular for its bilateralism and the circumstances of discovery.

## Patient and observation

Written informed consent was obtained from the patient for publication of this case report and any accompanying images. We present the case of mrs HK, 58 years old, rural origin, without any particular pathological history or breast cancer risk factors. She had 15 pregnancies and 15 parities. She has been in menopause for 8 years. Her disease history begun four months before her first consultation with a bilateral mammary out-flow and the retraction of the right nipple. These symptoms were ignored by the patient and the evolution was marked by a progressive atrophy of the two mammary glands, more accentuated at right, and thereafter with the development of ulcerations of the two breasts and a lymphoedema of the right arm added to a fever bringing the patient to medical consultation.

**Figure 1:**
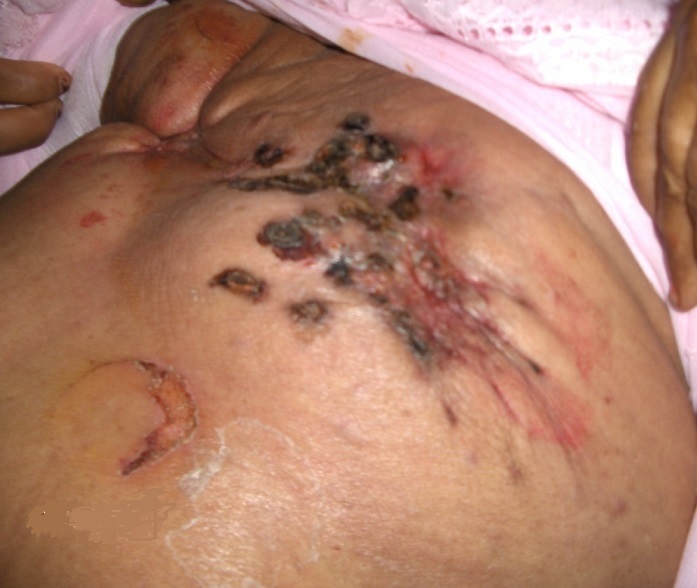
Atrophic scirrhus of the left breast

**Figure 2:**
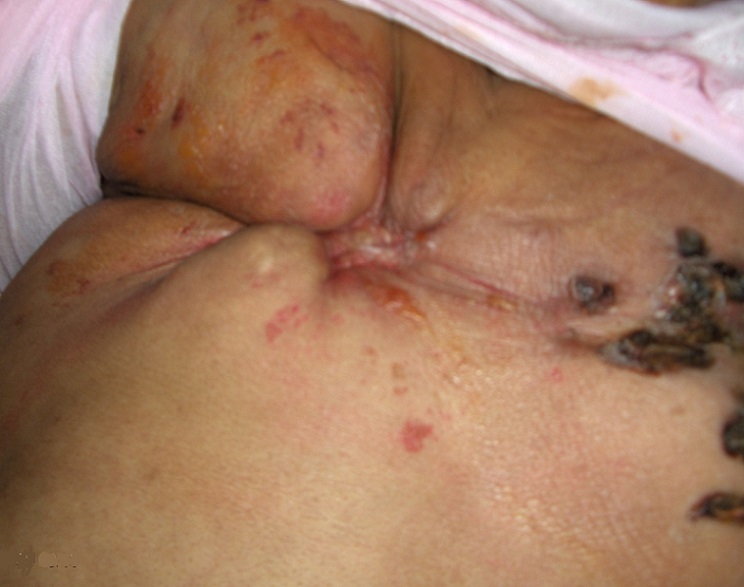
Atrophic scirrhus of the right breast

**Figure 3:**
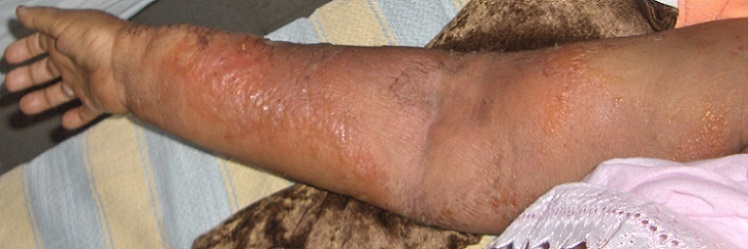
Lymphœdema of the right arm with erysipelas

The examination had shown an altered general condition marked by a septic shock, 40-degree fever with thrill, tachycardia and low blood pressure. The examination of the breasts, which is painful on palpation, revealed an atrophy of the two mammary glands which is more accentuated on the right side, associated to multiple permeation nodules, a cutaneous thickening and ulcerations of the two breasts (Figure 1, Figure 2). The examination of the lymph nodes areas had not shown any palpable adenopathy. The examination of the right arm had shown a lymphoedema with a hot blotchy plate similar to erysipelas (Figure 3).

The Doppler scan of the right arm had shown a tumourous process infiltrating the clavicular region and surrounding the veins. The blood cultures achieved on the admission were positive with the presence of streptococcus. The patient has been put under intra venous antibiotic therapy leading to a good clinical evolution. Some bilateral mammary biopsies have been achieved concluding to a bilateral infiltrating ductal carcinoma and positive hormonal receptors to the estrogens and to the progesterone. The extension analysis had shown with diffuse bone metastases and multiple liver secondary nodules. The rate of CA15-3 was of 81U/ml.

In terms of this extension assessment, the tumour was classified T4c bilateral N0 M1 according to the 2002 TNM classification of the American Joint Committee on Cancer staging system. The therapeutic decision was to start by a palliative chemotherapy of type FEC60, then, a hormonotherapy based on Tamoxifen. The evolution has been marked after 5 FEC cures by the progression of the illness to the left chest wall as well as the hepatic area with the ascension to the rate of CA15-3. Therefore chemotherapy of second line by Taxol has been indicated. However, in spite of the good clinical outcome (disappearance of the nodules with the decrease of the rate of CA15-3), this treatment was stopped at the end of the fourth cure because of the appearance of stern peripheral neuropathy requiring to put the patient under Tamoxifen and biphosphonate. The patient died eighteen months after her first consultation.

## Discussion

Breast cancer is the most common cancer among women [1]. Its incidence is increasing worldwide, but the prognosis is more improved by the installation of screening and educational programs and therapeutic advances. However, advanced breast advanced forms are still extant and reported in the literature [2, 3] due to some highly extensive histological forms or mostly to the retarded delay of consultation which can be explained by the lack of information women on the risk factors and signs suggestive of the disease and the problems of shame and modesty which remain an obstacle to the consultation especially in rural areas and economic hardship.

Breast carcinoma is the most common malignancy to metastasize to skin among women [4]. Hutchinson was the first to describe the cutaneous metastases of breast cancers. In 1893, he had described a case of cutaneous erythematous squirrhe associated with a mammary cancer. The erythematous infiltration was superficial and was followed by a blush and a light induration. The described lesion had recalled to the erysipelas, but there was a more important congestion attacking the sides of the erythema [5]. This form has also been observed in our patient called carcinoma “en cuirasse”. Mordenti *et al* [6] studied 164 cases of cutaneous metastasis from breast carcinoma. Clinical features included papules and /or nodules in 131 (80%), telangiectatic carcinoma in 19 (11.2%), erysipeloid carcinoma in five (three percent), carcinoma “en cuirasse” in five (three per cent), alopecia neoplastica in three (two per cent) and zosteriform pattern in one (0.8%).

En cuirasse metastatic carcinoma is characterized by diffuse morphea-like induration of the skin. It is a fibrotic process resembling the encasement in an armor of a curassiere (cavalry soldier) [7, 8], leading to a progressive retraction with disappearance of the mammary gland, then the ulceration plated on the costal grill. It evolves from firm papules and nodules overlying an erythematous base to a sclerodermoid plaque [9]. Pain and pruritus may be the associated features, unlike cutaneous metastases, which usually present as asymptomatic, painless, firm or doughy skin-colored papules or nodules. As we know, our observation corresponds to the first case of atrophic squirrhus en cuirasse carcinoma attacking the two breasts and being the reason of first consultation.

This advanced form of breast cancer is characterized by its being inoperable diagnosis and associated with a very limited survival rate. The therapeutic standard among these patients is a palliative medical treatment including symptomatic treatment, chemotherapy, hormonotherapy, and if possible followed by a surgery and/or local radiotherapy [10].

## Conclusion

The atrophic squirrhe of the breast appearing especially in aged women is a macroscopic term designating some varieties of carcinomas characterized by their high extensive potential, their retractable character related to the existence of an abundant fibrous stroma. The bilateral forms are much rarer. This case illustrates the lack of education and incites to widespread screening programs for breast cancer in rural tunisian population.
